# The Ecological Restoration Strategies in Terrestrial Ecosystems Were Reviewed: A New Trend Based on Soil Microbiomics

**DOI:** 10.1002/ece3.70994

**Published:** 2025-03-06

**Authors:** Yuanqi Zhao, Xiaojuan Yuan, Weiwei Ran, Zhibing Zhao, Di Su, Yuehua Song

**Affiliations:** ^1^ School of Karst Science Guizhou Normal University Guiyang China; ^2^ State Engineering Technology Institute for Karst Desertification Control Guiyang China

**Keywords:** climate change, karst, mine, restoration, soil microorganism, terrestrial ecosystem

## Abstract

Soil microorganisms play a pivotal role in the biogeochemical cycle and serve as crucial indicators of ecological restoration in terrestrial ecosystems. The soil microbial community is regarded as a pivotal participant in environmental processes, offering both positive and negative feedback to diverse media within the ecosystem. This community can serve as a potential indicator in ecological monitoring and restoration processes. Consequently, an increasing number of scholars are directing their research towards the field of soil microbial ecology in diverse ecosystems and fragile areas, with the aim of elucidating the intricate interactions between microbes and vegetation. However, the implementation of soil microbiome in ecological restoration remains in the experimental stage due to the interference of extreme events and the complexity of governance measures. Consequently, a comprehensive evaluation of existing research is imperative. This review aims to address the ecological crises currently experienced by diverse terrestrial ecosystems and to provide a comprehensive overview of the specific practices of soil microorganisms in the context of ecological restoration. We also incorporate them into fragile habitats and identify urgent issues that need to be addressed in the ecological restoration process of fragile areas.

## Graphical abstract

This article provides an overview of the ecological crises faced by different terrestrial ecosystems and discusses the contributions of soil microorganisms in addressing these crises. Overall, people pay more attention to the changes in soil microbial communities during vegetation succession and incorporate soil microorganisms as indicators into ecological assessment systems. However, the specific contribution of soil microorganisms in the restoration process is still in the experimental stage and has been applied to small‐scale habitats, such as soil remediation in farmland and mines.

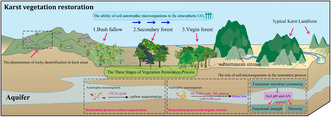

## 1 Introduction

The ecological problems caused by climate change continue to have negative impacts on terrestrial ecosystems (Krause et al. 2019). It is anticipated that more frequent disturbances, such as floods and droughts, will increase plant mortality and exacerbate ecological crises in fragile habitats (Seidl et al. 2017). Additionally, human activities, including overgrazing, deforestation, and mining, pose further threats to the stability of terrestrial ecosystems (Ostberg et al. 2018). These disturbances lead to landscape destruction and biodiversity loss, ultimately threatening human survival and health (Djuma et al. 2017). Consequently, ecological restoration efforts are economically and socially significant and remain a focal point in environmental research.

**FIGURE 1:**
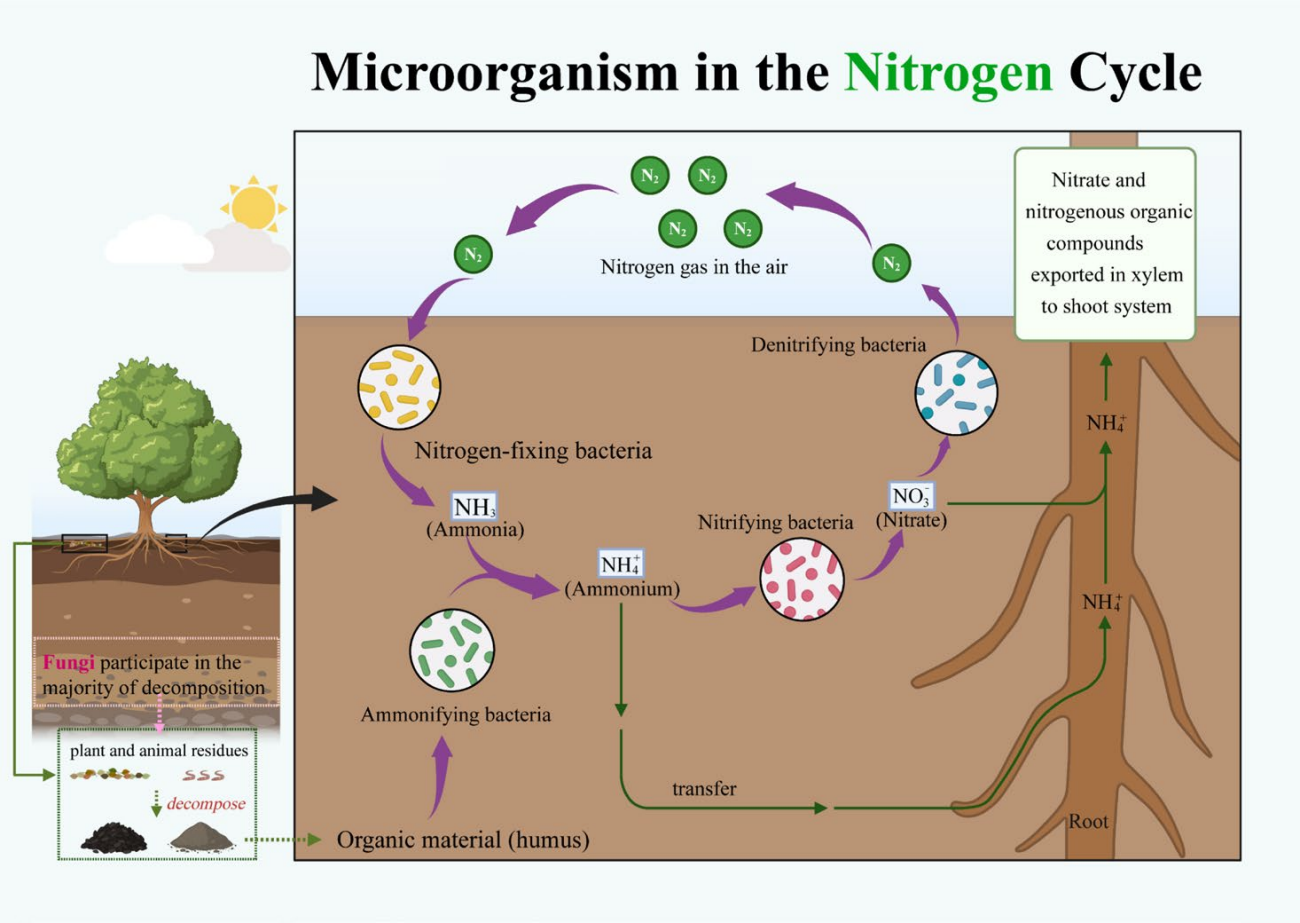
Microorganism participate in the nitrogen cycling processes in terrestrial ecosystems. Fungi break down organic matter into inorganic matter through the secretion of enzymes. This cycle example is highlighted in the bottom left corner of the picture, emphasizing the important process of fungi as decomposers participating in the material cycle.

At present, research on the restoration of terrestrial ecosystems mainly involves multiple dimensions such as environment (Hooper et al. 2016), economy (Carruthers et al. 2022), social culture (Robinson et al. 2021), and infrastructure (Zhang et al. 2023). Nowadays, facing the challenges of global climate warming and frequent extreme weather, more and more scholars are starting to search for feasible ecological restoration solutions from the perspective of soil microorganisms (Lara‐Severino et al. 2016; Bruni et al. 2023). Microorganisms play an important role in the soil sphere and are important drivers of biogeochemical cycles (Figure 1), affecting the material cycling and energy flow of different terrestrial ecosystems (forests, grasslands, deserts, etc.) (Van Der Heijden et al. 2007).

According to Li et al. (2022), soil microorganisms have a significant role in the process of plant succession and are intimately associated with the roots of vegetation. As molecular biology technology continues to advance, multi‐omics combined techniques, including metabolomics and genomics, have progressively helped to clarify the mechanism of interaction between plant roots and soil microorganisms (Liu et al. 2022; Zhong et al. 2024). According to a growing body of research, microbial inoculants and soil autotrophic microorganisms are crucial for ecological restoration, ecosystem function improvement, soil carbon sequestration, and soil function enhancement (Nowak et al. 2015; Laanbroek et al. 2022). Meanwhile, studies of soil microbes in environmentally vulnerable regions are steadily growing (Li et al. 2020; Shi et al. 2022). Their low environmental carrying capacity and complex spatial heterogeneity cannot withstand large‐scale engineering remediation. Vegetation succession, functional microorganisms, and microbial inoculants can improve regional environmental pollution and ecological degradation problems.

In summary, there is a necessity to comprehensively understand and review the ecological crisis that terrestrial ecosystems are currently experiencing, the specific measures for soil microbial communities in the process of ecological restoration, and to focus on specific measures for microbiome involvement in ecological restoration in ecologically fragile areas, especially in karst ecosystems and mining ecosystems with complex human land conflicts, exploring the feasibility of incorporating microbial technology into their ecological restoration. The following is a detailed list of the content:

1. *Elaborated on the ecological crises faced by different terrestrial ecosystems*;
2. *Summarized the specific practices of soil microorganisms participating in the ecological restoration of terrestrial ecosystems*;
3. *Summarized the main trends in research related to karst ecosystems and mining ecosystems*;
4. *Feasible microbial remediation technologies for karst and mining ecosystems have been identified*.

**FIGURE 2:**
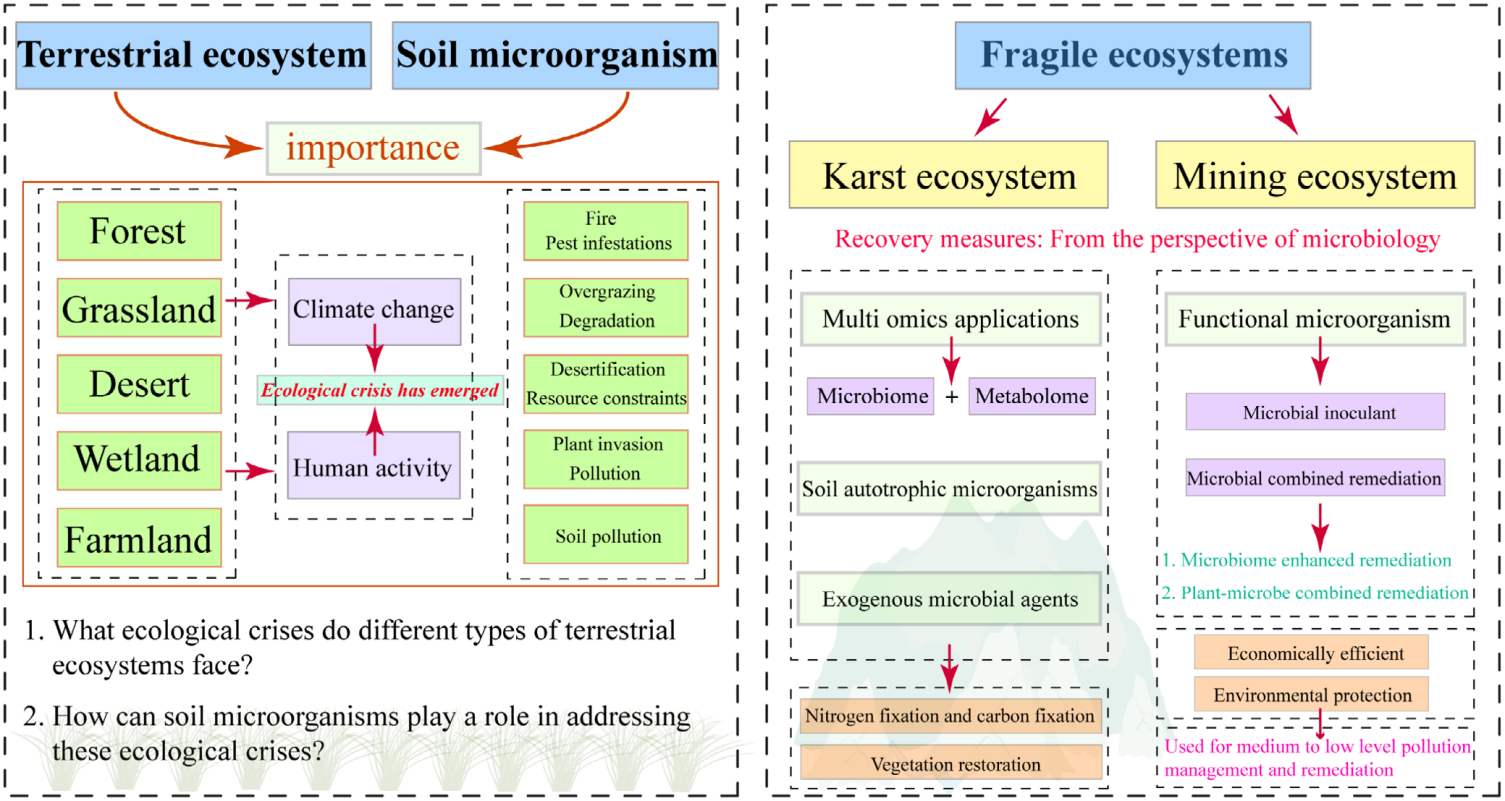
The main structure and content of this review.

The keywords for database search were: microbial ecology, soil microbiome, terrestrial ecosystem, karst rocky desertification, ecological restoration, and community. The main database is Web of Science (http://webofknowledge.com). Please refer to Figure 2 for the main guide of this article.

## 2 The Importance of Incorporating Soil Microbial Communities Into the Ecological Restoration of Terrestrial Ecosystems

Microorganisms play a pivotal role in the formation of plants and the biogeochemical processes within soil ecosystems, as highlighted by Zuo et al. (2023). Furthermore, during the formation and development of aboveground vegetation, soil microorganisms exhibit diverse life strategies and provide environmental feedback (Rebi et al. 2024). Aboveground vegetation demonstrates robust plasticity in the context of ecological restoration, significantly influencing the stability of ecosystems (Lisboa et al. 2021). However, when ecosystems confront severe environmental stressors, ecologists prioritize not only the conservation but also the restoration of degraded habitats as a crucial step toward enhancing ecosystem services and resilience (Rawat et al. 2023).

**FIGURE 3:**
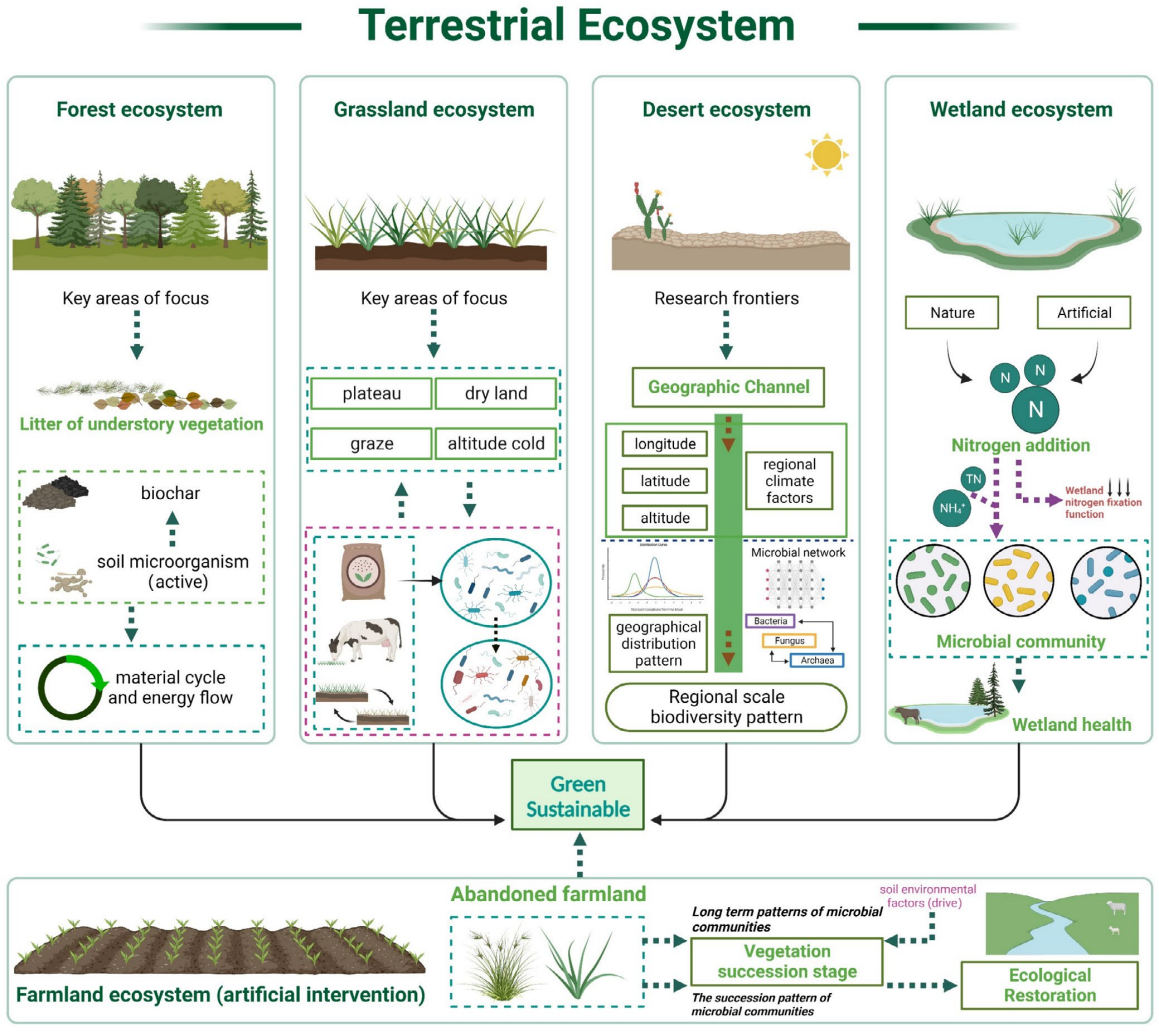
Current hot research topics in different types of terrestrial ecosystems (partial). The figure only shows representative hotspots for terrestrial ecosystem restoration work.

**FIGURE 4:**
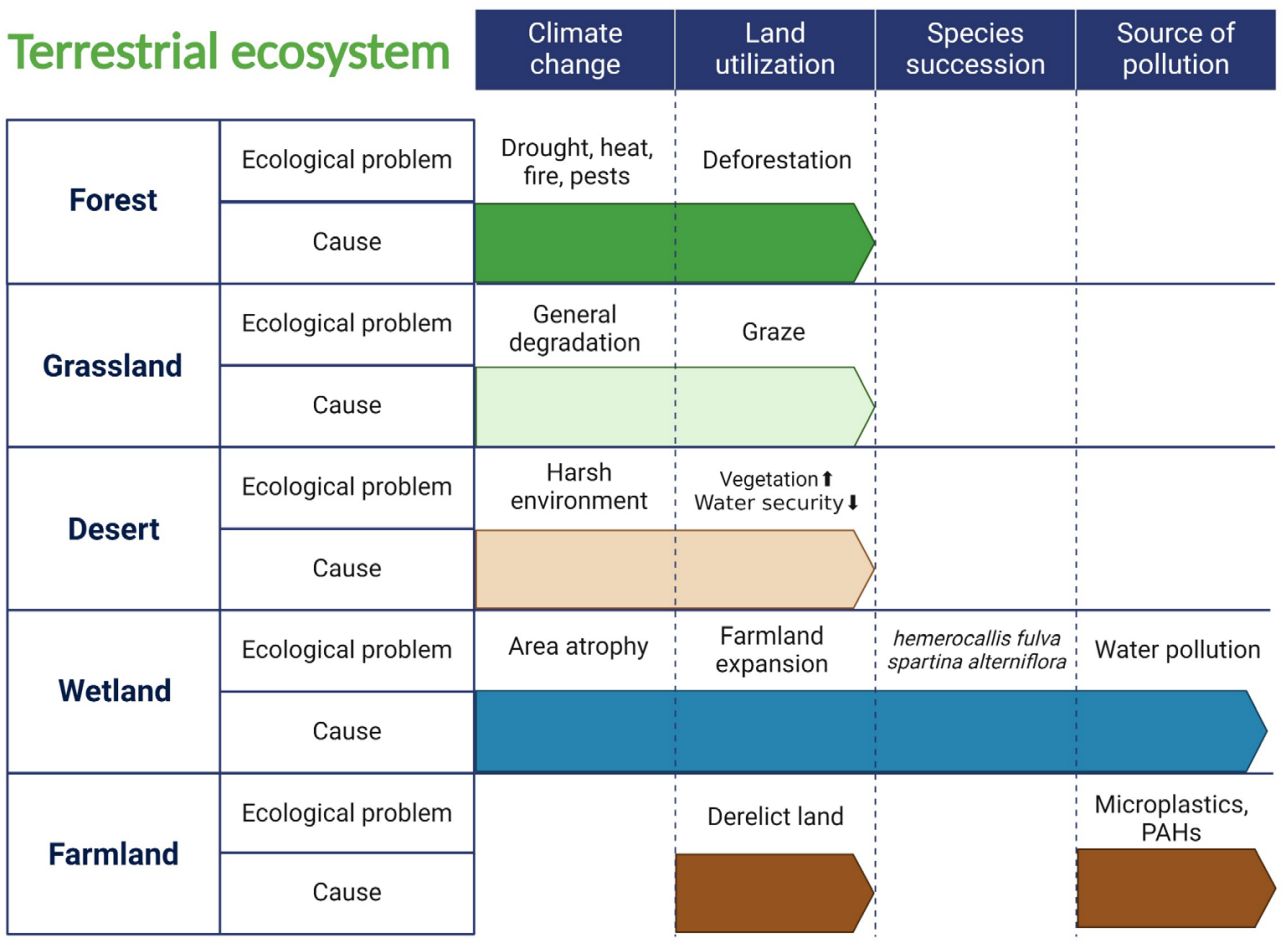
Ecological crises and causes faced by different terrestrial ecosystems. Cause includes aspects Climate change, Land utilization, Species succession, Source of pollution, etc.

In ecological restoration efforts, the rejuvenation of aboveground vegetation often takes precedence. Notably, the establishment of vegetation communities exerts a profound impact on the pedosphere, with which they are intimately associated (Ri et al. 2020), thereby facilitating the reestablishment of animal and microbial communities. Although this may not be a universal phenomenon, it underscores the necessity for biologists to effectively shape microbial communities to catalyze ecological recovery. This is because the nutrient cycles that sustain the plant life cycle are heavily reliant on soil microorganisms (Sun et al. 2024). It is well documented that maintaining the turnover of matter and energy in the biosphere is a fundamental function of soil microorganisms (Dobrovol'skaya et al. 2015), playing a crucial role in the evolution of terrestrial ecosystems (Chen et al. 2016). With the continuous advancement and sophistication of molecular biology techniques, an increasing number of ecologists are inclined to consider soil microorganisms as ideal indicators for assessing the versatility and ecological restoration of terrestrial ecosystems (Figures 3 and 4).

### 2.1 Characteristics of Typical Terrestrial Ecosystems and Ecological Crises

#### 2.1.1 Forest Ecosystems Are Facing Many Ecological Crises Caused by Climate Change

The biggest terrestrial ecosystem, forests offer economic, ecological, and social advantages (Wu et al. 2022). However, as a result of climate change and biodiversity loss, the structure and function of forest ecosystems are increasingly becoming out of balance (Moomaw et al. 2019). This is partly due to the development of agriculture and land use associated with population and urbanization (Ahrends et al. 2010). Global weather and climate patterns are shifting, and the atmospheric circulation is anomalous as a result of global warming (Franzke and Sentelles 2020). Climate change in East Asia will cause the intensification of the East Asian summer monsoon circulation, intensify thermal forcing across the Qinghai‐Tibet Plateau, increase the frequency of extreme weather events, and significantly affect the biological balance of forest ecosystems (Cleavitt et al. 2021). For instance, mountain floods brought on by exceptionally high rainfall will reduce the soil in forest ecosystems (Breuning‐Madsen et al. 2017), decrease the amount of vegetation and soil layer beneath forests (Ochoa‐Cueva et al. 2015), exacerbate the degree of rocky desertification, and worsen the state of the ecosystems. Furthermore, local drought episodes brought on by shifts in the global atmospheric circulation would indirectly raise the frequency of forest fires (Hu et al. 2021). The Australian fire cycle has been prolonging in recent years, partly due to climate change, and it is worth mentioning that the Midwest area of Australia is a natural “fire spot” every summer. In addition to the significant disturbances imposed on forest ecosystems by fires indirectly attributed to climate change, the phenomenon of forest mortality resulting from prolonged drought and elevated temperatures stands as a direct manifestation of the adverse impacts of climate change and warrants serious attention (Anderegg et al. 2013). This issue underscores the urgent need to consider the multifaceted effects of climate change on forest health and resilience.

#### 2.1.2 Grasslands Face Many Challenges From Grazing and General Degradation Processes

Grassland occupies one‐fifth of the world's land area, providing immeasurable ecological and economic benefits for human society (Yang et al. 2020a). The primary production sensitivity grasses exhibit towards climatic variations within terrestrial ecosystems is noteworthy (Knapp and Smith 2001). Concurrently, changes in global climate, nitrogen deposition, and land use profoundly affect processes and functions within grassland ecosystems (DeCock et al. 2023). Grassland degradation represents negative formalization regarding its ecological characteristics being an ongoing process (Bardgett et al. 2021). Each stage during degradation faces different environmental challenges (Yao et al. 2016); dynamic control over grassland degradation along rational ecological restoration has become a significant challenge requiring immediate attention globally. Unreasonable overgrazing has been identified through numerous studies as the main human activity leading towards grassland degradation (Zhu et al. 2023). Grazing, unlike forests, impacts both directly and indirectly upon grasslands (Schroeder et al. 2025); grasslands having relatively single species and nutrient structures compared with forests makes their environmental carrying capacity lower, thus making them more susceptible towards human activities (Liu et al. 2024a; Niu et al. 2024). Some studies indicate grazing affects not only vegetation but also physical and chemical properties alongside microorganisms present within soils, causing land degradation alongside desertification occurring from mineral exploitation and grassland reclamation, accelerating these processes.

#### 2.1.3 A Highly Stable Taxon in a Desert Environment Is the Key to Ecological Restoration

Arid environments and desert ecosystems contain a variety of potential resources (Keshtkar et al. 2016), and although their biodiversity is much lower than that of forest and grassland ecosystems, some studies indicate that deserts cover about 35% of the Earth's land area and have significant carbon reserves (Jia et al. 2023). Water security has always been a key ecological issue in desert ecosystems (Abdullah et al. 2022). Some studies have pointed out that although the ecological restoration plan (ERP) has achieved good results in soil conservation and sand fixation, the expansion of vegetation will intensify the competition for limited water resources in the region and affect regional water security (Xu et al. 2023). What is clear is that revegetation may not work in desert areas. On the one hand, desert ecosystems are faced with various external pressures such as extreme water shortage, high radiation, soil carbon and nitrogen limitation, and nitrogen deposition (Chen and Costanza 2024); on the other hand, the vegetation restoration process will consume scarce water resources in desert ecosystems, and the transpiration of plants will cause water to disperse into the atmosphere, thus forming a vicious circle and aggravating desertification. As such, ecologists are focusing more on groups that can be more stable in desert ecosystems, such as microbes, in search of more viable ecological restoration strategies.

#### 2.1.4 Plant Invasion: The Arduous Task of Wetland Ecological Restoration

Different from other ecosystems, the wetland ecosystem has the highest productivity, extensive environmental functions, and ecological benefits (Chatanga et al. 2020; Das et al. 2022). Global climate change and human activities have caused many wetland areas to shrink and environmental pollution to be serious (Luo et al. 2020; Mahdian et al. 2024). The researchers involved project that (Blankespoor et al. 2014) around 64% of freshwater marshes, 72% of coastal wetlands, and 61% of brackish/saline wetlands in 86 developing nations would be at risk from a 1 m sea level rise brought on by climate change. At the same time, wetlands are vulnerable to alien species invasion (Yang et al. 2020b). In recent years, there have been many reports of alien species invasion in the global wetland ecological crisis, like *Hemerocallis fulva* (Sennikov and Lazkov 2022) and *Spartina alterniflora* (Wang and Lin 2023). Plant invasion destroys the vegetation diversity of wetlands and faces an incalculable ecological degradation crisis (Tesitel et al. 2020). Invasive species compete for regional resources (Harris et al. 2020) and change the biological community and the physical and chemical properties of soil. As an effective participant in soil processes, soil microorganisms are highly sensitive to changes in vegetation communities. Similarly, soil microorganisms are equally important in the study of heavy metal migration and redistribution mechanisms (Zhang, Liu, et al. 2024). Therefore, whether it is plant invasion or wetland pollution, ecologists have gradually taken soil microorganisms as an important reference for wetland ecological restoration.

#### 2.1.5 Major Problems Facing Farmland Ecosystems

The traditional model of agricultural development has resulted in land degradation, soil pollution (Nawab et al. 2021), and an increase in abandoned farmland (Wang et al. 2020). With the development of the economy in recent years, polycyclic aromatic hydrocarbons (PAHs) are widely present in soil related to the petroleum industry (Mastral and Callen 2000). Due to their potential toxicity and carcinogenicity, PAHs have become the focus of agricultural soil pollution research. Studies have shown that PAHs affect soil function and quality and ultimately endanger human health through all levels of the food chain (Wilcke 2007). In recent years, ecologists have carried out soil remediation through traditional physical and chemical techniques (Mu et al. 2024), but due to high cost and damage to soil structure and ecosystem function to a certain extent, more researchers have begun to apply bioremediation in line with the development model of modern agriculture (Sun et al. 2018). For example, using efficient bacteria, bioremediation has been used to treat PAH‐contaminated soils. In earlier years, researchers successfully remediated PAH‐contaminated soil with alfalfa and revealed the PAHs degrading potential of rhizosphere microbial communities (Muratova et al. 2003). It is of concern that microplastics produced in the process of agricultural production also pose a great threat to the current farmland ecosystem (Kumar et al. 2020), and the mechanism of their impact on ecological processes is still unclear, so clarifying the impact of microplastics on soil ecosystems has become an important issue for ecologists to solve. Different from soil pollution, abandoned farmland can maintain secondary succession (active or passive), and the succession process can rebuild vegetation communities, which has certain significance for the reshaping of ecosystem functions and soil characteristics (Wang et al. 2018). At present, researchers are placing more emphasis on the revegetation of abandoned farmland and emphasizing the soil processes closely linked to it (Wang et al. 2020). With the continuous development of metagenomics and single‐cell sequencing technology, soil microorganisms are becoming more and more important in the process of farmland ecological restoration (Enebe and Babalola 2021; Tian, Zheng et al. 2024). For example, a large number of microorganisms are attached to the surface of microplastics (Tian, Wang et al. 2024). Relevant studies will have a profound impact on soil pollution control.

### 2.2 Microbial Communities Play an Important Role in Solving Ecological Crises

As previously indicated, extreme weather events, including high temperatures, droughts, and rainstorms, which are increasingly prevalent due to climate change, have led to an escalation in forest disturbances and mortality (Reis et al. 2022). These disturbances, in turn, have resulted in the degradation of forest ecosystems (McColl‐Gausden et al. 2022).

#### 2.2.1 Soil Microorganisms Widely Participate in the Process of Vegetation Reconstruction

In the context of ecological restoration, soil plays a pivotal role in both natural and artificial succession processes (Strickland et al. 2017). Vegetation restoration measures have been shown to have a profound impact on degraded soil ecosystems (including, but not limited to, soil carbon and nitrogen cycles, soil water retention, and other soil physical properties) (Chen, Xu et al. 2024). The role of soil microorganisms in the process of vegetation restoration has been demonstrated through empirical evidence, and their application in the ecological restoration of degraded forests has been steadily increasing. A recent study indicated that Ascomycota, Basidiomycota, and Glomeromycota showed potential for improving soil fertility in degraded *Pinus massoniana* forests (Wang et al. 2024a). Another study identified substantial disparities in biomarkers between bacterial and fungal communities in undisturbed forests, artificial larch forest belts, and fire sample plots, with fungal communities exhibiting greater stability or resistance to disturbance compared to bacterial communities (Zhai et al. 2023). Furthermore, during secondary forest succession, changes in the fungal communities from Ascomycota to Basidiomycota reflect the growing accumulation of soil nutrients and the maturation of the ecosystem (Chai et al. 2019). In light of these findings, ecologists are increasingly focusing their research on the dynamics of plant features during forest succession (Mawa et al. 2020). Soil abiotic qualities (i.e., its physical and chemical properties) represent the primary elements that link plant features to soil microorganisms (Lamizadeh et al. 2019). The process of plant community succession is initiated when initial plant‐induced alterations in nutrient availability facilitate the development and establishment of subsequent plants. A substantial body of evidence indicates that plant traits may serve as a pivotal driver of the interrelationship between plant and soil abiotic properties and microbial communities (Ma et al. 2024). Furthermore, shifts in specific microbial groups during succession have the potential to modify the environment‐driven patterns of plant traits and other traits at the species level (Pohl et al. 2011). The alterations in soil microbial communities have been demonstrated to influence plant traits, particularly those related to leaf and root characteristics (Laughlin 2011).

#### 2.2.2 Reducing the Impact of External Stress on Ecosystems Can Be Achieved Through Microbial Community Interactions

Ecological degradation caused by drivers such as overgrazing and climate change affects nearly half of the world's grasslands (Dlamini et al. 2014; Gibbs and Salmon 2015). It is well known that grassland degradation can reduce soil microbial diversity, change community structure (Yu et al. 2021), and affect grassland ecosystem function (Ren et al. 2021). However, microbial communities are highly structured, and the interactions of different taxa between communities can be represented by network structures. In general, network communities with a high degree of complexity are more stable (Metelmann et al. 2020). Some studies have pointed out that the complex interaction of different groups in soil microbial communities can enhance the ability of ecosystems to resist extreme climates (Bardgett and Caruso 2020). For example, some ecologists have pointed out that climate warming and other weather disturbances can change the structure (Shi et al. 2020) and stability of soil microbial communities and then affect a series of soil ecological processes such as the soil carbon cycle (Wang et al. 2024b). It has been suggested that grassland degradation may reduce the complexity and stability of soil fungal communities and ecosystem versatility, and that the effects of this degradation may persist through the initial stages of grassland restoration (Luo et al. 2023). Meanwhile, when degraded patches form in grassland systems, the proportion of nitrifiers, plant pathogenic fungi, and saprotrophic fungi in the soil increases significantly, which increases the risk of nitrogen leaching and plant diseases (Che et al. 2019). Thus, it is not conducive to ecological restoration. In the process of grassland restoration, some scholars have pointed out that the addition of biochar and effective microorganisms (EM) is conducive to the rapid recovery of degraded ecology (Li et al. 2023). On the one hand, biochar provides more habitats for saprophytic fungi, and EM can promote root growth (Dangi et al. 2020), which is conducive to the growth of saprophytic fungi. On the other hand, saprophytic fungi can accelerate the decomposition of litter or dead roots, improve soil fertility, and improve the absorption of mineral elements by vegetation roots, thus improving the tolerance and disease resistance of plants to harsh environments (Cao et al. 2022).

#### 2.2.3 Microorganisms Are Ideal Objects for Ecological Restoration in Extreme Environmental Areas

Due to urbanization expansion and the global food crisis, cultivable land is insufficient to meet the current needs of human society. In recent years, more agricultural practitioners have planned to include desert areas in their agricultural land use plans (Perera et al. 2018). Microbes have great potential to adapt to extreme environments (Moreno et al. 2008), so researchers have incorporated microbes into the restoration of desert ecosystem ecology. Soil fungi and bacteria may shape plant diversity in different ways (Yang et al. 2021). Bacteria can enhance diversity by improving the environment with functional strains, while fungi can limit diversity through pathogen attack. A recent study has shown that (Chen et al. 2024) the bacterial functional network in desert areas is more stable than in ecological ecotones, and an increase in the relative abundance of some functional genes may be harmful to nitrogen cycling and vegetation growth. Moss is often the primary biological surface cover of desert ecosystems (Belnap et al. 2016). Like soil microorganisms, its particular properties allow it to adapt to harsh desert settings (Adessi et al. 2021). Desert ecosystem processes have been altered as a result of the severe bryophyte crust degradation catastrophe that has affected most desert places across the world. As previously said, the use of microbial resources for ecological restoration has grown increasingly popular, and bryophyte crust restoration is no exception. It has been demonstrated that *Bacillus megaterium* and *Actinomyces bovis* greatly enhance soil structure and nutrient availability, encouraging moss development and metabolism by increasing energy generation (Ji et al. 2017). Research indicates that the most successful method exhibited a large number of protonemata, which eventually developed into moss and enhanced its density and coverage (Zhou et al. 2018); this is mainly attributed to the increase of exogenous microorganisms. Analogous to mosses, microalgae and cyanobacteria exhibit remarkable adaptability to unique ecological niches, even thriving under the harshest weather conditions prevalent in desert landscapes. An earlier study highlighted the significant role of nitrogen‐fixing bacteria in bolstering soil fertility and promoting algal diversity, particularly featuring species such as *Chlamydomonas* sp., *Chlorococcum humicola* , and *Chlorella vulgaris* . This process has demonstrated marked improvements in soil texture, enhancing nutrient accessibility and organic matter content within desert ecosystems, ultimately fostering an optimal habitat conducive to the establishment of desert soil and the proliferation of cryptogams. Consequently, this phenomenon plays a pivotal role in reinstating and enhancing biodiversity within these arid regions (Li et al. 2003).

## 3 Soil Microorganisms Can Do More in Ecological Restoration Work

As mentioned earlier, when ecosystems face external threats such as climate change and human activities, they will experience varying degrees of ecological degradation. The self‐maintenance and self‐repair of ecosystems will accelerate the process of ecological restoration, which is an ideal state. However, some ecosystems have unique geological backgrounds (Mahapatra et al. 2018) and developmental histories (Fang et al. 2022) that result in low environmental carrying capacity and susceptibility to external disturbances, leading to weak ecological restoration capabilities, long cycles, and increasingly severe ecological problems (Chen, Zhang et al. 2024). For example, soluble rocks such as limestone are widely distributed in southwestern China, and the abundant hydrothermal conditions accelerate their dissolution, forming typical karst landforms through long‐term external and internal forces (Xiong et al. 2002). Due to the long process of limestone soil formation, abundant water and heat conditions in the region, and serious soil erosion problems, vegetation degradation and ecological problems such as rocky desertification have occurred (Zhang, Xiong, et al. 2020). At the same time, the karst region in southwestern China has a large population, and the special ecosystem is difficult to maintain its own stability (Yuan and Li 2023). Ecological problems such as soil erosion and rocky desertification are becoming increasingly severe, seriously hindering ecological protection and economic development. Unlike the poor maintenance capacity of karst ecosystems, mines, as important energy sources in the industrialization process, play a crucial role in traditional industries such as heavy industry (Omotehinse et al. 2020). However, with the depletion of mineral resources and the increasing number of abandoned mines, the environmental pollution problems (especially soil pollution) they bring are becoming increasingly severe, leading to a decrease in the adaptability of the ecosystem and causing losses to the ecological welfare of future generations of humanity (Xiang et al. 2021; Cubley et al. 2022). Therefore, it is necessary to review the ecological restoration work carried out on fragile karst ecosystems and abandoned mine ecosystems in order to provide ideas for scientifically improving the self‐sustaining capacity of ecosystems.

**FIGURE 5:**
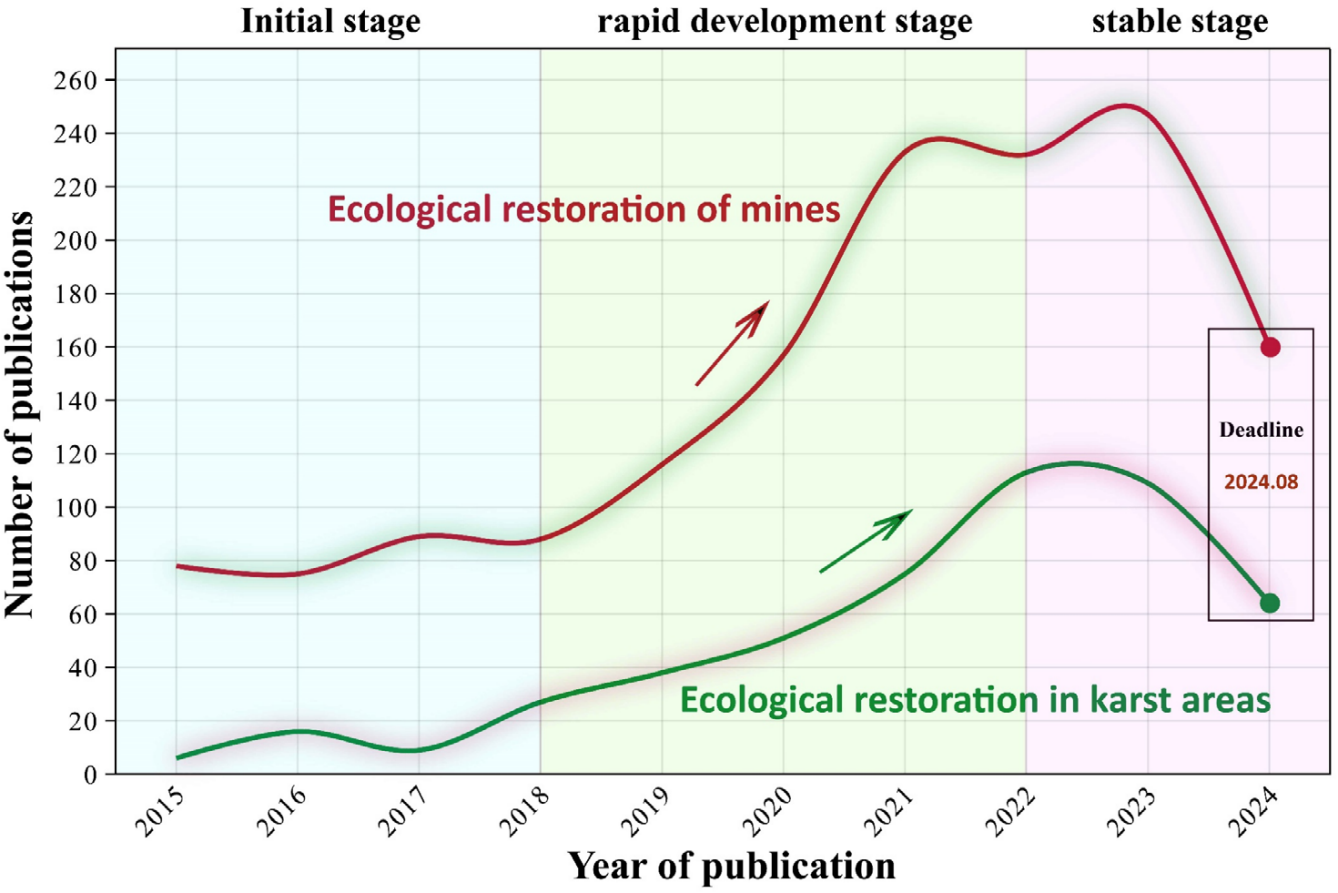
Trend in the number of research papers published on karst ecosystems and mining ecosystems in the past decade. Based on Web of Science database search, the search time range is from January 2015 to August 2025.

In order to identify research trends in related fields, we follow the systematic literature review (SLR) method, drawing on the logic and systematicity of SLR (van Dinter et al. 2021), with the aim of using a transparent and boundary‐defining approach to help us identify the main trends in our research direction. In order to better present the research progress trends of karst ecosystems and mining ecosystems, we strictly set keywords according to the research boundaries during the retrieval stage. Simultaneously, the search will be conducted in the form of intersecting main directions and research directions, with the search date starting from January 1, 2015. The specific keyword settings are as follows: karst + ecological restoration, mining + ecological restoration. Literature statistics show that retrieval targets based on two directions exhibit roughly the same trend (Figure 5); among them, the period from 2018 to 2022 is a period of rapid development in two major fields, with a rapid increase in the number of publications.

### 3.1 Microbial Multi Omics Application Is Key to Karst Ecological Restoration

**FIGURE 6:**
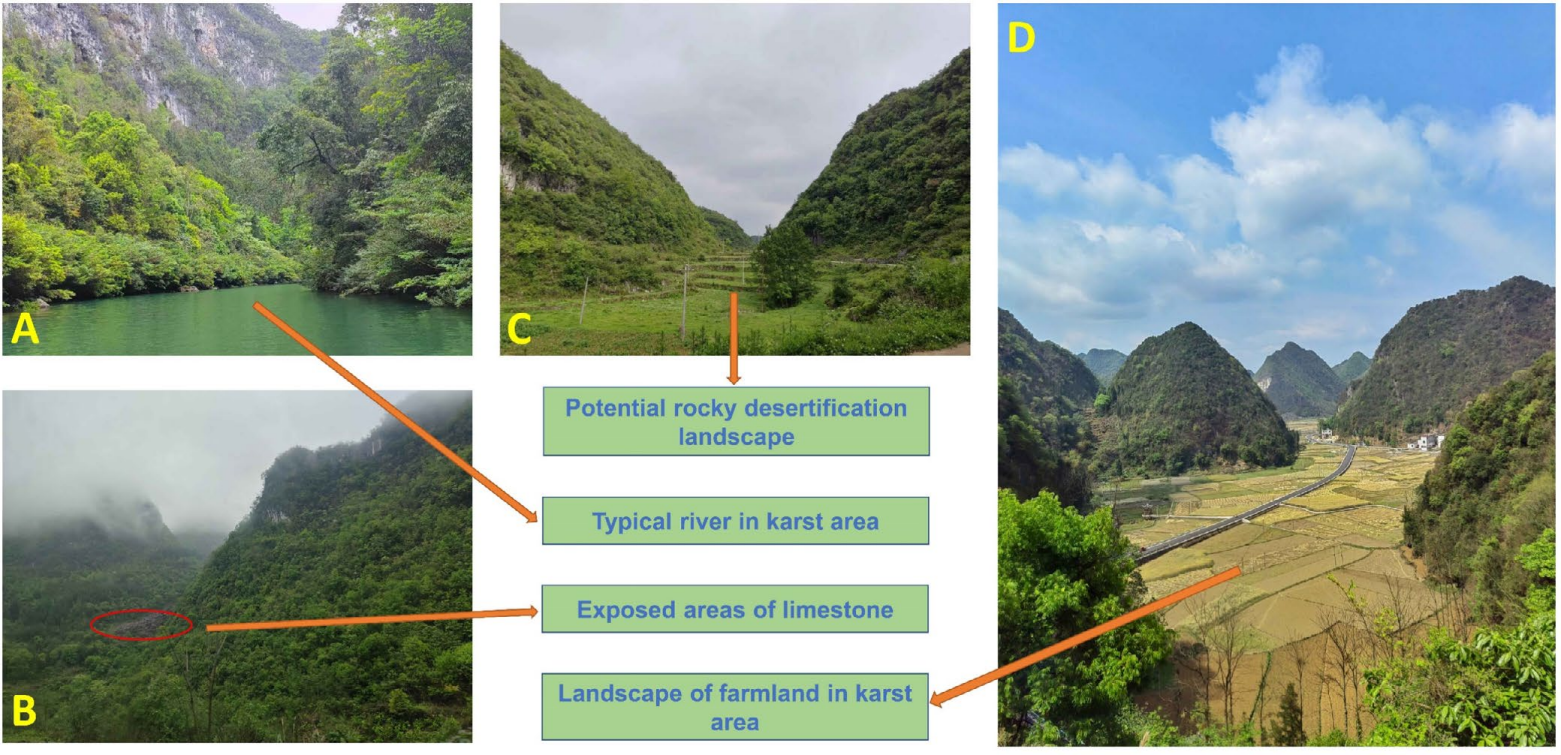
Representative landscapes in karst ecosystems. The above pictures were taken by the author in 2023–2024. Among them, (A) is located in Zhangjiang, Libo County, Guizhou Province, China; (B) is located in Libo County, Guizhou Province, China; (C) is located in Salaxi Town, Qixingguan District, Bijie City, Guizhou Province; (D) is located in Huishui County, Guizhou Province.

The karst ecosystem is a paradigmatic example of a fragile ecosystem, characterized by significant challenges related to soil erosion and rocky desertification (Kaufmann 2014). The ecological restoration of such environments adheres to the principles of secondary succession, as illustrated in Figure 6. Karst vegetation succession is an ecological phenomenon characterized by predictable changes in plant communities. These changes can serve as indicators of environmental changes (Qi et al. 2013). Concurrently, vegetation succession exerts an influence on the composition of both aboveground and underground ecosystems, including, but not limited to, the composition of soil microorganisms (Tian, Zhang et al. 2024). Consequently, in the course of vegetation restoration, researchers have begun to regard soil microbial communities as a pivotal indicator of ecological restoration (Papik et al. 2023). However, it should be noted that a single microbiome does not provide a comprehensive representation of the mechanisms by which microbial communities or specific species participate in soil processes. Consequently, it cannot propose efficient ecological restoration strategies (Zhong et al. 2024). Studies have demonstrated that the process of vegetation succession can result in alterations in soil function and metabolism (Zhong et al. 2024). The metabolic distribution and function of vegetation communities at different stages of succession are different, and these metabolites can alter soil ecology and respond to environmental demands (Tang et al. 2024). The types and abundance of soil metabolites are influenced by soil microorganisms, plants, and environmental factors. Consequently, this comprehensive understanding facilitates the evaluation of soil quality, biodiversity, and metabolic activity. Moreover, it enables a more profound comprehension of the impact of vegetation succession on soil metabolism, particularly in ecologically fragile karst regions facing crises such as rocky desertification (Li et al. 2023).

In current research, some scholars have found that fatty acids undergo significant changes during the secondary succession process of karst vegetation, which means that changes in cell membrane composition may be a key difference between microorganisms in different vegetation stages (Zhong et al. 2024). It is worth noting that unsaturated fatty acids can regulate the transformation and release of nutrients by soil microorganisms, further optimizing soil fertility and crop nutrient absorption. When people try to improve the productivity of degraded karst wetlands, researchers have found that planting rice will increase the rate at which active microorganisms in the soil convert inorganic carbon into organic carbon (Wang et al. 2022). The degradation of natural wetlands will reduce the carbon sequestration potential of karst wetlands, while agricultural reclamation will maintain the nitrogen fixation potential of karst wetlands (Kang et al. 2024). *Bradyrhizobium* is one of the main carbon‐fixing microorganisms in karst wetland soil and is a dominant microbial community in reclaimed karst wetlands (Wang et al. 2021). It can be considered to be incorporated into land productivity restoration work in the form of live microbial preparations.

**FIGURE 7:**
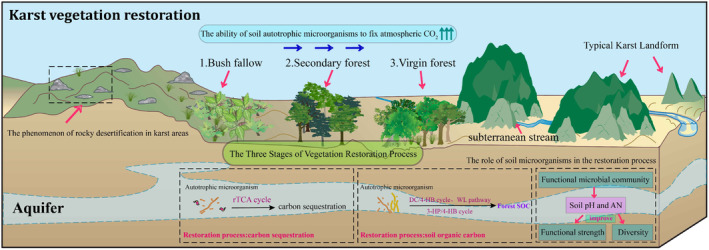
The role of soil microorganisms in vegetation restoration in karst ecosystems. Biological carbon sequestration pathway: rTCA, reductive tricarboxylic acid cycle; DC/4‐HB, Dicarboxylic acid/4‐hydroxybutyric acid; WL pathway, Reductive acetyl CoA pathway; 3‐HP/4‐HB, 3‐hydroxypropionic acid/4‐hydroxybutyric acid.

Autotrophic microorganisms in soil play an important role in ecosystems, unlike exogenous microbial agents (Akinyede et al. 2020). In a variety of habitats, autotrophic microorganisms play a critical role in the fixation of atmospheric CO2 and the sequestration of soil organic carbon (SOC) (Akinyede et al. 2020). Prior studies employing DNA‐SIP technology have highlighted the pervasive presence of autotrophic microorganisms in karst soils (Li et al. 2018). A recent study suggests that during the vegetation restoration process of degraded karst forests (Figure 7), when the restoration stage transitions from shrub forests to secondary forests, there is a significant change in the fixation of atmospheric CO2 by soil autotrophic microorganisms, leading to an increase in microbial diversity and functional intensity (Dai et al. 2024). It is imperative to acknowledge that when assessing the efficacy of functional microbial community recovery, it is essential to prioritize not only the diversity and abundance of these communities but also to acknowledge the significance of microbial functional genomics, encompassing functional strength and potential (Cong et al. 2015).

### 3.2 A Powerful Assistant for Ecological Restoration in Mines: Functional Microorganisms

Coal mining has a long history dating back thousands of years (Hu et al. 2015). It has significantly impacted the natural environment even if it has greatly increased societal output (Bashirzadeh et al. 2022). According to Palmer et al. (2010), overexploitation destroys ecosystems, hydrology, and landscapes; puts endangered species in jeopardy; and lowers biodiversity. According to Bruneel et al. (2019), mining operations have the potential to negatively impact ecosystem services and functions by causing surface subsidence, air pollution, soil pollution, and water pollution. Microbial ecosystems will also be significantly impacted by the open‐pit mining method (Howladar 2016). Vegetation restoration is a popular and cost‐effective way to restore damaged land in coal mining sites, just as other ecosystems employ ecological restoration (Zhang, Zhang, et al. 2024). Long‐term plant restoration can greatly enhance soil quality and soil microbial community variety in coal mining regions, according to several recent research studies (Zverev et al. 2022). To reduce the succession time and increase the stability of the soil microbial ecology, physical, chemical, or biological amendments are frequently combined in soil reclamation projects (Zhao et al. 2019). This is due to the fact that the regeneration of soil microbial communities is just as important to the effective reclamation of mine land as the growth of flora (Ohsowski et al. 2012).

#### 3.2.1 Microbial Inoculants

In traditional concepts, people generally pay attention to the composition and diversity of soil microorganisms during vegetation restoration (Ramos‐Tapia et al. 2022). Traditional vegetation restoration has a long succession cycle and is greatly affected by climate fluctuations (Zhang et al. 2020). Some countries and regions often use foreign and rapidly growing herbs and shrubs in mining area reclamation (Cubley et al. 2022), which leads to the suppression of local species and may prevent natural succession in the area. Numerous studies have shown that ecological restoration through vegetation competition reduces plant diversity (Jeong et al. 2024), thereby affecting soil microbial diversity. Therefore, in order to reduce fluctuations in ecosystem stability, the use of mixed planting or sowing with local species has been increasing in mining area development. Meanwhile, microbial inoculation is a promising method for restoring disturbed terrestrial ecosystems (Andjelkovic et al. 2020; Caillon et al. 2021). As mentioned earlier, *Bradyrhizobium* is one of the main carbon‐fixing microorganisms in karst wetland soils (Wang et al. 2021) and can be considered a form of active microbial agent incorporated into land productivity restoration work. Arbuscular mycorrhizal fungi (AMF) are a type of mycorrhizal fungi that can be introduced into degraded soils to improve soil properties and vegetation growth (Ma et al. 2023). They are important microbial inoculants in the field of ecological restoration. In the soil affected by subsidence mining areas, AMF inoculation can promote plant absorption of effective nutrients, especially nitrogen acquisition, and increase plant soil consolidation capacity, thereby reducing soil erosion and desertification (Bi et al. 2019). Although microbial inoculants are widely used in soil improvement and ecological restoration projects, research mainly focuses on their specificity and function, rather than the process of constructing and restoring dominant populations and their survival characteristics (Jia et al. 2022). In fact, soil microorganisms, with their vast quantity and diversity, can quickly respond to environmental changes. Therefore, it is crucial to apply an appropriate amount of active cells in areas that require inoculation to resist adverse growth environments (Lu et al. 2024).

#### 3.2.2 Microbial Combined Remediation

Here, we highlight a joint ecological restoration system that aims to offer suggestions for mine ecological restoration (Lu et al. 2024). Microbiome‐enhanced remediation is a microbial combination approach that uses many microorganisms or their metabolites to heal damaged habitats or improve environmental quality, in contrast to microbial inoculants (Jia et al. 2022). The synergistic effect of two or more microorganisms is typically involved in microbial combined remediation in the soil remediation process (Song et al. 2022). These microorganisms can be functional microorganisms isolated and cultured in the laboratory (Yang et al. 2020c) or native microorganisms that were initially present in the soil. In certain environments, a joint remediation of various microorganisms that occupy distinct ecological niches and have the ability to work in concert may decrease the possible adverse effects of microbial communities (Vick et al. 2019), boost the diversity and turnover of core microbial communities, lower the energy needed by soil to perform specific metabolic functions, and speed up nutrient accumulation and remediation processes (Atuchin et al. 2023). Microbial‐assisted plant remediation technology, which is a crucial method of ecological restoration, differs from microbiome‐enhanced remediation in that it uses the properties of plant roots to introduce particular pollutant‐degrading and plant‐promoting bacteria into plants (Yang et al. 2020d; Li et al. 2024) (Table 1). Lin et al. (2021) emphasized the important regulatory role of microorganisms in plant remediation of soil heavy metal pollution. Root‐associated microorganisms can improve soil remediation by altering plant nutrient composition and reducing heavy metal toxicity.

**TABLE 1:** Application of plant microbial combined remediation.

Plant	Microbial inoculant	Combined effect	References
*Salix viminalis*	*Crucibulum laeve* strain	Degradation of PAHs	Ma et al. 2020
*Triticum aestivum*	Bacterial consortium (BDAM)	Degradation of Bispyribac Sodium	Ahmad et al. 2019
*Sedum alfredii*	*Bacillus* sp.M6	Reduce the bioavailability of Cd	Abid et al. 2022
*Vallisneria natans*	*Enterobacter ludwigii*	Reduce Cd pollution in sediments	Liu et al. 2024b
*Stenotrophomonas rhizophila* SPA‐P69	*Zea mays* L.	Improved plant health and increased beneficial bacterial phyla	Kusstatscher et al. 2020
*Dolichos lablab*	Bacterial consortium (BDAM)	Degradation of bensuluron	Zhang et al. 2022

Microbial remediation technology has undoubtedly advanced in treating soil contamination (Li et al. 2024), but research on habitat community building, plant regeneration, and other topics is comparatively lacking. However, the majority of microbial combination technologies have not yet been extensively used and are still in the experimental stage (Hu and He 2020). Microbial remediation techniques can be given priority for ecological restoration in regions with considerable soil heterogeneity, such as habitat fragmentation and coal mining zones. Microbial remediation is appropriate for application in delicate ecosystems because, in contrast to other bioremediation techniques, it is highly safe, inexpensive, and environmentally benign (Shi et al. 2022).

## 4 Conclusions and Future Prospects

This article presents a comprehensive overview of the ecological crises confronting diverse terrestrial ecosystems and examines the pivotal role of soil microorganisms in addressing these challenges. Currently, there is a growing emphasis on monitoring changes in soil microbial communities during vegetation succession, with soil microorganisms increasingly being utilized as indicators within ecological assessment frameworks. However, despite these advancements, the precise contribution of soil microorganisms to the restoration process remains in the experimental phase and has primarily been applied to smaller‐scale habitats, such as soil remediation in agricultural and mining contexts.

The ecological degradation of terrestrial ecosystems poses a global challenge that necessitates the adoption of rational and effective measures. Based on the distinct characteristics of soil microorganisms involved in soil processes and existing practices, we propose the following initiatives: (1) Implement vegetation restoration plans while closely monitoring the dynamic shifts in soil microbial communities in tandem with vegetation succession patterns; (2) Introduce rhizosphere‐associated functional microorganisms to bolster the soil‐stabilizing capabilities of plants in erosion‐prone areas; (3) Investigate the carbon sequestration mechanisms of soil autotrophic microorganisms, including nitrifying and sulfur‐oxidizing bacteria, to gain deeper insights into the role of soil microorganisms in soil organic carbon accumulation and their implications for climate change; (4) Employ microbial remediation technologies, including microbial combinations and plant‐microbial synergies, to tackle soil pollution and ameliorate ecological degradation.

## Author Contributions

**Yuanqi Zhao:** conceptualization (equal), data curation (equal), formal analysis (equal), investigation (equal), methodology (equal), software (equal), validation (equal), visualization (equal), writing – original draft (equal). **Xiaojuan Yuan:** conceptualization (equal), investigation (equal), supervision (equal), validation (equal), writing – review and editing (equal). **Weiwei Ran:** investigation (equal), methodology (equal), software (equal), supervision (equal), writing – review and editing (equal). **Zhibing Zhao:** methodology (equal), supervision (equal), writing – review and editing (equal). **Di Su:** supervision (equal), validation (equal). **Yuehua Song:** conceptualization (equal), funding acquisition (equal), investigation (equal), methodology (equal), project administration (equal), resources (equal), supervision (equal), writing – review and editing (equal).

## Ethics Statement

The authors have nothing to report.

## Consent

The authors have nothing to report.

## Conflicts of Interest

The authors declare no conflicts of interest.

## Data Availability

The authors have nothing to report.
